# Biosynthesis of Natural Rubber: Current State and Perspectives

**DOI:** 10.3390/ijms20010050

**Published:** 2018-12-22

**Authors:** Xiao Men, Fan Wang, Guo-Qiang Chen, Hai-Bo Zhang, Mo Xian

**Affiliations:** 1 CAS Key Laboratory of Biobased Materials, Qingdao Institute of Bioenergy and Bioprocess Technology, Chinese Academy of Sciences, No.189 Songling Road, Laoshan District, Qingdao 266101, China; menxiao@qibebt.ac.cn (X.M.); wangfan@qibebt.ac.cn (F.W.); chengq@qibebt.ac.cn (G.-Q.C.); 2 University of Chinese Academy of Sciences, Beijing 100049, China

**Keywords:** natural rubber biosynthesis, rubber particles, *cis*-prenyltransferase (CPT), *Hevea* rubber transferase 1 (HRT1), rubber elongation factor (REF), small rubber particle protein (SRPP), HRT1-REF bridging protein (HRBP), *Hevea brasiliensis*

## Abstract

Natural rubber is a kind of indispensable biopolymers with great use and strategic importance in human society. However, its production relies almost exclusively on rubber-producing plants *Hevea brasiliensis*, which have high requirements for growth conditions, and the mechanism of natural rubber biosynthesis remains largely unknown. In the past two decades, details of the rubber chain polymerization and proteins involved in natural rubber biosynthesis have been investigated intensively. Meanwhile, omics and other advanced biotechnologies bring new insight into rubber production and development of new rubber-producing plants. This review summarizes the achievements of the past two decades in understanding the biosynthesis of natural rubber, especially the massive information obtained from the omics analyses. Possibilities of natural rubber biosynthesis in vitro or in genetically engineered microorganisms are also discussed.

## 1. Introduction

Rubber is a kind of basic necessities widely used in human society. It can be divided into synthetic rubber and natural rubber according to its source. Synthetic rubber are polymers of alkenes or dienes that come from petrochemical industry. Natural rubber are biopolymers from rubber-producing plants, and have unique properties which include resilience, elasticity, abrasion and impact resistance, efficient heat dispersion, and malleability at cold temperatures that cannot be matched by synthetic rubber [1]. As a result, natural rubber are irreplaceable in many applications (heavy-duty tires, medical devices, surgical gloves, etc.). According to International Rubber Study Group’s (IRSG) report, the global natural rubber consumption was 13.2 million tons with a corresponding value of 26 billion US dollars in 2017 and showed an increasing tendency.

There are more than 2500 rubber-producing plants from various taxa distributed throughout the plant kingdom. Most of these plants have unfavorable characteristics, such as a very low yield of rubber or low molecular weight of the polymer [2]. The Para rubber tree *Hevea brasiliensis* is the almost exclusive species producing commercially viable natural rubber. However, due to its specialized requirements for growth conditions, susceptibleness to fungal infections and laborious harvest work, new rubber-producing species which can be grown in colder and less productive geographical areas, such as Russian dandelion (*Taraxacum kok-saghyz*), guayule (*Parthenium argentatum* Gray) and hardy rubber tree (*Eucommia ulmoides*), have been domesticating for industrial rubber production purpose [2,3,4]. *E. ulmoides* is one of the few woody plants capable of producing abundant quantities of *trans*-polyisoprene in their roots, barks, leaves and fruits, even from young *E. ulmoides* trees [5,6]. Both *T. kok-saghyz* and *P. argentatum* are annual or perennial *Asteraceae* herbs that have short maturation time and can be planted and harvested mechanically. Rubber extracted from *P. argentatum* latex has the similar molecular weight but much fewer allergic proteins than that from *H. brasiliensis* [1,4]. In 2015, the first tire made of *P. argentatum* latex was produced by Bridgestone Corporation and exhibited great potential in future tire market.

Although many progresses have been made in finding more suitable candidates for industrial rubber production purposes, the inefficiency of the new species is still the obstacle. Progress made in omics analyses and the established *Agrobacterium*-mediated genetic transformation of these rubber-producing plants would accelerate the improvement of their rubber yields [7,8,9,10]. In addition, the relatively simple genome and short life cycle also made *T. kok-saghyz* an ideal model system for studying rubber biosynthesis [11]. 

In the past two decades, outstanding issues in natural rubber production, such as rubber polymerase identification, polymerization mechanism and molecular weight determination, have been investigated intensively. In this review, we briefly summarize the recent progresses in natural rubber biosynthesis process, the involved genes/proteins and pathways, rubber particle biogenesis, and the useful clues obtained from omics data, which would be helpful for understanding natural rubber biosynthesis and high-yielding rubber-producing plants breeding. We also discuss the possible alternative source of natural rubber besides the rubber-producing plants.

## 2. Biosynthesis of Natural Rubber and the Involved Genes/Proteins

### 2.1. Biosynthesis and Physiological Roles of Natural Rubber

**Figure 1:**
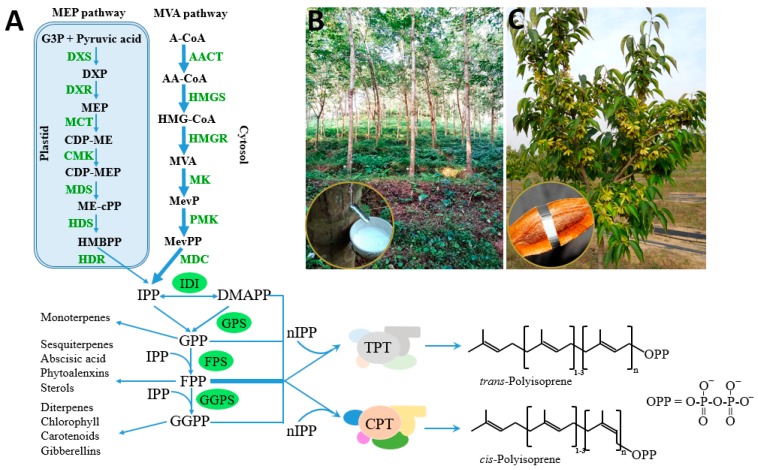
Biosynthesis of natural rubber and *trans*-polyisoprene and the related isoprenoid pathway. (**A**) Biosynthetic pathway of natural rubber and *trans*-polyisoprene, and the related 2-C-methyl-D-erythritol-4-phosphate (MEP), mevalonate (MVA) and the oligomeric allylic pyrophosphates synthetic pathway, “n” depends on species [2]; (**B**) the exclusively commercial *cis*-polyisoprene producing plant—Para rubber tree *Hevea brasiliensis*, with the lower left picture showing rubber tapping; (**C**) the *trans*-polyisoprene producing plant—hardy rubber tree *Eucommia ulmoides*, with the lower left picture showing *trans*-polyisoprene containing in its fruit [5]. Intermediates in MEP pathway: G3P, glyceraldehyde-3-phosphate; DXP, 1-deoxy-d-xylulose-5-phosphate; MEP, 2-C-methyl-d-erythritol-4-phosphate; CDP-ME, 4-(cytidine-5′-diphospho)-2-Cmethyl-d-erythritol; CDP-MEP, 2-phospho-4-(cytidine-5′-diphospho)-2-C-methyl-d-erythritol; ME-cPP, 2-C-methyl-d-erythritol-2,4-cyclodiphosphate; HMBPP, 4-hydroxy-3-methylbut-2-enyl diphosphate; IPP, isopentenyl pyrophosphate; DMAPP, dimethylallyl pyrophosphate. Enzymes in MEP pathway: DXS, DXP synthase; DXR, DXP reductoisomerase; MCT, CDP-ME synthase; CMK, CDP-ME kinase; MDS, ME-cPP synthase; HDS, HMBPP synthase: HDR, HMBPP reductase; IDI, IPP isomerase. Intermediates in MVA pathway: A-CoA, acetyl-CoA; AA-CoA, acetoacetyl-CoA; HMG-CoA, hydroxymethylglutaryl-CoA; MevP, mevalonate-5-phosphate; MevPP, mevalonate pyrophosphate; IPP, isopentenyl pyrophosphate; DMAPP, dimethylallyl pyrophosphate. Enzymes in MVA pathway: AACT, A-CoA C-acetyltransferase; HMGS, HMG-CoA synthase; HMGR, HMG-CoA reductase; MK, mevalonate kinase; PMK, MevP kinase; MDC, mevalonate pyrophosphate decarboxylase. GPP, geranyl pyrophosphate; GPS, geranyl pyrophosphate synthase; FPP, farnesyl pyrophosphate; FPS, farnesyl pyrophosphate synthase; GGPP, geranylgeranyl pyrophosphate; GGPS, geranylgeranyl pyrophosphate synthase; TPT, *trans*-prenyltransferase; CPT, *cis*-prenyltransferase.

**Figure 2:**
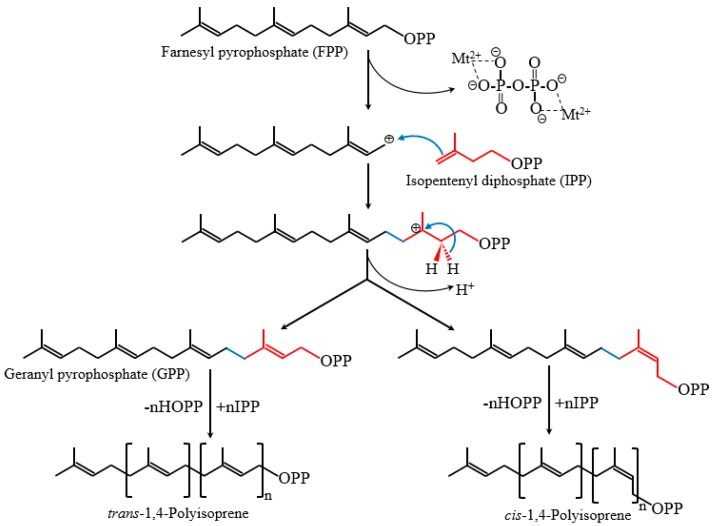
Proposed initiation and polymerization mechanism in rubber biosynthesis.

Natural rubber consists mainly of *cis*-1,4-polyisoprene, of which the constituent monomer isopentenyl pyrophosphate (IPP) is synthesized from the mevalonate (MVA) pathways and possibly also from the 2-C-methyl-d-erythritol-4-phosphate (MEP) pathway in plants (Figure 1). Its biosynthesis is catalyzed by rubber transferase [EC.2.5.1.20] and involves three steps: initiation, polymerization, and termination. Rubber chain initiation needs an oligomeric allylic pyrophosphates initiator which could be dimethylallyl pyrophosphate (DMAPP), geranyl pyrophosphate (GPP), farnesyl pyrophosphate (FPP), geranylgeranyl pyrophosphate (GGPP) or *cis*-allylic pyrophosphate in vitro [1,12]. However, the low binding constant of FPP compared with other initiators, the *trans*-*trans*-*cis* sequence detected by NMR at the initiating terminal of rubber molecules, and the cytosolic location of FPP suggested that FPP is the main initiator in vivo [1,13,14,15]. The rubber transferase needs divalent cations, such as Mg^2+^ or Mn^2+^, as “activity cofactors” and the elementary IPP incorporation process is very similar to those of cationic polymerization [13,16,17,18]. Initiation starts by an enzyme-divalent cationic cofactor-assisted ionization of the carbon–oxygen bond of the initiator and yields an allylic cation plus pyrophosphate counteranion [19] (Figure 2). Subsequently, the vinylidene group of IPP adds to the allylic carbocation yielding a tertiary carbocation, which, via proton elimination, regenerates an allylic pyrophosphate end [19]. With the continuously incorporation of IPP monomers, the rubber molecules grow as a “living carbocationic polymerization” and the *cis*-*trans* stereoregulation depends on specific rubber transferases which generate certain type of double band after IPP addition and proton elimination [19]. The termination of the polymerization, or in other words the molecular weight determinant, remains unclear. Natural rubber exhibits broad molecular weight distribution and species-specific molecular weight difference, indicating that multiple factors may be involved besides certain regulatory proteins. In vitro rubber biosynthesis assay revealed that the concentration of FPP and IPP and the ratio of them affect the rubber biosynthetic rate and rubber molecular weight. Increasing IPP concentrations lead to increased rubber molecular weight, whereas increasing FPP concentrations have the opposite effect [20]. Magnesium concentrations were also demonstrated to regulate substrate affinity and rubber molecular weight in vitro [13,16]. In addition, through recent latex proteome analyses, the termination process was proposed to involve an ubiquitin-proteasome-mediated proteolysis system for protein degradation [21]. However, the detailed mechanism needs to be further investigated.

Natural rubber seems like a “dead-end product” because it remains inside plant till its death and no enzyme that could degrade latex was found in plants. Then why would plants convert so much matter and energy into rubber biosynthesis? The physiological role of latex and rubber molecules remains a mystery. Latex was supposed to have functions in wound healing, disease-resistance and stress-tolerance, because defense and stress-related genes expand in the genome of rubber-producing plants and are expressed abundantly in latex compared with in other tissues [22,23,24]. Some studies demonstrated a lot of chemicals and proteins in the latex are toxic to herbivores and latex may capture and immobilize the mouth parts of insects due to its sticky nature [25,26]. In *P. argentatum* and *H. brasiliensis*, which have no isoprene synthase to eliminate excess photosynthate, rubber was suggested to serve as an excess assimilate sink to prevent damage to the photosynthetic apparatus under conditions of cold temperature, high light or other environmental stimuli [23,27]. Additionally, signaling molecules such as ethylene and jasmonates, which play a major role in plant stress regulation, can induce laticifer differentiation and transient increase in latex production, also implying the involvement of latex in stress responses [28].

### 2.2. Structure, Components and Biogenesis of Rubber Particles

The exact tissues where natural rubber is synthesized are different, depending on plant species. For example, rubber synthesis happens in specialized laticifer in phloem of *H. brasiliensis*, *T. kok-saghyz* and *E. ulmoides*, whereas it takes place in gutta-containing cells derived from epithelial cells in parenchyma tissue and pith in *P. argentatum* [2,24,29,30]. Through ultracentrifugation, latex can be separated in at least 3 compartments: the cream with rubber particles and Frey-Wissling particles, the cytoplasmic C-serum and the bottom fraction with lutoids [31]. Natural rubber biosynthesis happens on the surface of rubber particles which is one of the main components of latex (30–50% by volume in *H. brasiliensis*) [32]. Rubber particles are spherical or pear-shaped subcellular organelles that have diameter ranges of 0.02–3.0 μm in *H. brasiliensis*, 1.0–2.0 μm in *P. argentatum*, 0.2–1.0 μm in *T. kok-saghyz*, 1.6–6.5 μm in *Ficus* species, and 0.02–1.2 μm in *Euphorbia characias* [1,33,34,35,36,37]. They are made of polyisoprene, surrounded by a monolayer of lipids with proteins and other compounds [1]. Proteins involved in rubber synthesis associate with or were inserted into the lipidic monolayer and were thought to form a complex or work synergistically during the rubber biosynthesis [31,38]. The synthesized polyisoprene chains were elongated inward and stored in the interior of the rubber particles, resulting in the enlargement of the rubber particles. Besides, proteins involved in stress resistance and rubber flocculation, allergic proteins and function unknown proteins were also found on rubber particles [37,39,40,41].

Rubber particles were supposed to originate from endoplasmic reticulum (ER) or Golgi according to studies of their structure and the proteins isolated from the rubber particles, which have typical ER or Golgi localization such as rubber elongation factor (REF), small rubber particle protein (SRPP), *cis*-prenyltransferase-like protein (CPTL), clathrin and Rab GTPases [1,39,42,43,44,45]. This view was also supported by the findings that phosphatidylcholine, phosphatidylethanolamine and glycoproteins are abundant in the monolayer biomembrane, which is typical for the cytoplasmic part of the ER bilayer membrane [46]. It was postulated that after the first polyisoprene chain was synthesized by the rubber transferase complex localized on the ER, it was inserted into the hydrophobic region between the lipid bilayer. When more polyisoprene chains were synthesized, the region formed a convex outward with budding from the ER into the cytosol, and was finally separated from the ER membrane and generated the rubber particles [1,29,47,48]. 

Rubber particles can be classified into small rubber particles (SRPs) and large rubber particles (LRPs) [35]. Besides the size difference, SRPs were demonstrated to have much higher rubber biosynthesis activity than LRPs by in vitro assays, which may be due to the different protein profiles between them [37,49,50]. Moreover, the stored natural rubber molecules inside the rubber particles were supposed to have different architectures between SRPs and LRPs. SRPs are mainly composed of linear molecules with no or few branches, while LRPs contain branched molecules with join points originating from phospholipids [51].

### 2.3. Genes/Proteins Involved in Natural Rubber Biosynthesis

#### 2.3.1. *cis*-Prenyltransferase

**Figure 3:**
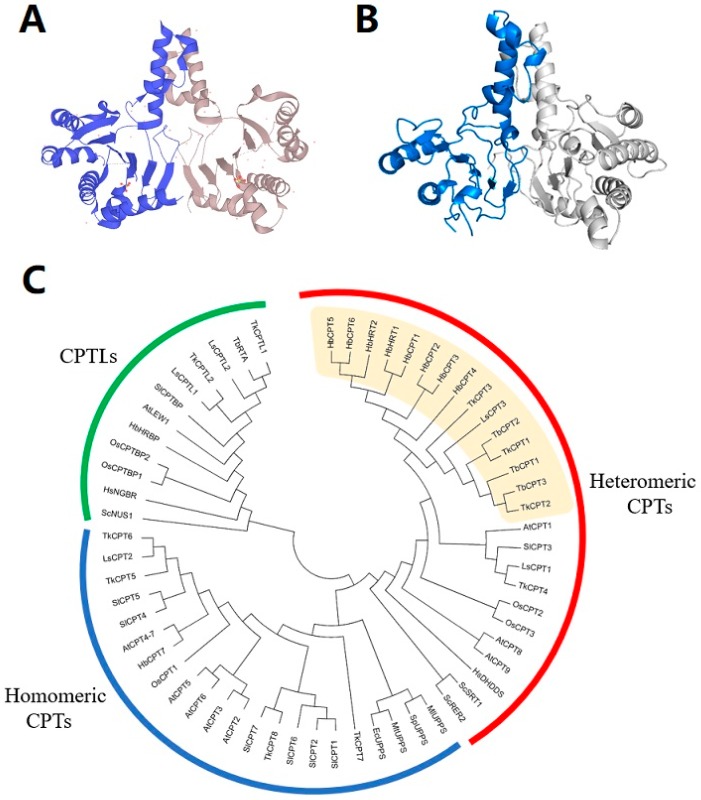
Three dimensional (3D) structure and phylogenetic analysis of CPTs and CPT-Like proteins (CPTLs). (**A**) Crystal structure of *Micrococcus luteus* undecaprenyl diphosphate synthase (MlUPS) homodimer (UniProtKB-O82827). (**B**) 3D structure of HbHRT1 (gray)-HbHRBP (blue) heterodimer predicted by PyMOL. (**C**) Phylogenetic analysis of CPTs and CPTLs. A total of 60 CPTs/CPTLs sequences were collected from human, plants and microbes, and the Neighbor-Joining (NJ) tree was constructed by MEGA6.0. Three clades are presented in the NJ tree: 1) the homomeric CPTs which catalyze the biosynthesis of short-chain and medium-chain prenols, including bacterial CPTs and plastidial CPTs; 2) the heteromeric CPTs which catalyze the biosynthesis of dolichol or natural rubber, including cytosolic CPTs from yeast, plants, and human; 3) the CPTLs which interact and form heterodimer with CPTs. The light orange part indicates CPTs involved in natural rubber biosynthesis. HbCPTs and TkCPTs/TbCPTs are separated into two subclades, indicating the polyphyletic origins of CPTs involved in natural rubber biosynthesis. Species abbreviations are: *At*, *Arabidopsis thaliana*; *Ec*, *Escherichia coli*; *Hb*, *Hevea brasiliensis*; *Hs*, *Homo sapiens*; *Ls*, *Lactuca sativa*; *Ml*, *Micrococcus luteus*; *Mt*, *Mycobacterium tuberculosis*; *Sc*, *Saccharomyces cerevisiae*; *Sl*, *Solanum lycopersicum*; *Sp*, *Streptococcus pneumoniae*; *Os*, *Oryza sativa*; *Tb*, *Taraxacum brevicorniculatum*; *Tk*, *Taraxacum kok-saghyz*.

Genes/proteins involved in rubber synthesis were investigated for nearly 50 years. In 1969, Archer and Cockbain first suggested that rubber transferase from rubber particles was responsible for natural rubber biosynthesis [52]. In the following studies, researchers revealed that the rubber transferase should be a protein complex consisting of several subunits. The *cis*-prenyltransferases (CPTs), which belong to the prenyltransferase family, were demonstrated to have the activity of incorporating IPP into the polyprenyl chains and were thought to be the most likely candidate or at least one component of the rubber transferase. CPTs were cloned and characterized from both prokaryotes and eukaryotes, and were proved to catalyze the synthesis of dolichols which is important for protein glycosylation [53,54]. They can be divided into two major classes: i) prokaryotic type including bacterial CPT and plastidial CPT; ii) eukaryotic type including cytosolic CPT from yeast, plants, and animals [55,56] (Figure 3). The crystal structure of prokaryotic CPTs was characterized and they were proved to form homodimers, while the eukaryotic CPTs were demonstrated to be heteromeric enzymes composed of at least two heterogeneous subunits [55,57,58].

Asawatreratanakul et al. cloned two CPTs from *H. brasiliensis* latex and designated them as HRT1 and HRT2 (*Hevea* rubber transferase) [59]. HRT1 and HRT2 share 87.3% identity of amino acid sequences, and both of them have all the five highly conserved regions which are important for catalytic function and substrate binding for *cis*-prenyl chain elongating enzymes [59,60]. HRT1 and HRT2 are expressed predominantly in the latex and may be unstable cytosolic proteins when expressed independently [44,59]. Enzyme activity assay of recombinant protein produced from *E. coli* showed that only HRT2 could catalyze the formation of long-chain polyprenyl products with approximate sizes of 2 × 10^3^–1 × 10^4^ Da in the absence of washed bottom fraction particles, while both recombinant HRT1 and HRT2 expressed from eukaryotic cell systems showed polyisoprenoids-producing activity with chain lengths of C_80–100_ without centrifuged latex fractions, indicating that post-translational modifications or latex-specific co-factors may be required to enable rubber transferase activity of HRT1/HRT2 [59,61]. In addition, three CPTs were cloned and characterized from *T. brevicorniculatum* [62,63]. The deduced amino acid sequences of TbCPTs showed ~98% identity with each other and were conserved at a lower level with *H. brasiliensis* CPTs (~53% identity with HRT1, ~52% with HRT2) [62]. They were functionally analyzed by complementary expression in yeast, and were demonstrated to catalyze the synthesis of *cis*-polyprenol dedol-PP [33]. The TbCPTs RNAi lines exhibited a significant reduction in rubber biosynthesis, fewer and smaller rubber particles and a complete loss of in vitro long-chain prenyltransferase activity of whole latex protein, indicating that rubber biosynthesis in *T. brevicorniculatum* latex is fully dependent on CPT activity [63]. Four deduced CPT-like protein sequences similar to an *H. brasiliensis* CPT (AAR88763.1) were identified in expressed sequence tag (EST) collection of cold-acclimated *P. argentatum*, but so far no independent enzymatic activity assay of PaCPT has been performed [64].

#### 2.3.2. Rubber Elongation Factor and Small Rubber Particle Protein

The rubber elongation factor (REF) and small rubber particle protein (SRPP) are homologous proteins originating from a common ancestor and belong to the stress-related protein superfamily [31]. They are the most abundant components of the rubber particle proteome [39,40]. The 14.7 kDa HbREF (138 aa) and 22.3 kDa HbSRPP (204 aa) were cloned and investigated even earlier than HbCPTs [65,66,67,68,69]. They are the main allergens of latex proteins, so were also designated as Hevb1 and Hevb3, respectively [31]. Isoforms of REF and SRPP were identified and all REF/SPRR proteins have a similar ~110 aa region called REF domain. REF subfamily members have a variable N-terminal and a relatively short C-terminal beyond the REF domain, while SRPP subfamily members have a short N-terminal and a variable C-terminal [70]. SRPPs are localized abundantly on SRPs, while different REF isoforms were found, either locating mainly on LRPs or expressing equally on both SRPs and LRPs [37,71,72]. HbREF interacts with HbSRPP, and was bound to the rubber particle lipidic monolayer more tightly than HbSRPP [31,44]. Both of them have aggregative properties and may function in rubber particle stabilization and coagulation [71,73].

Both REF and SRPP were reported to be required for the natural rubber biosynthesis. They were demonstrated to have the activity of promoting the incorporation of IPP into whole latex or washed rubber particles, and this activity could be inhibited by the corresponding antibodies [67,69,74]. RNAi silencing lines of REFs or SRPPs often showed reduced latex content and rubber transferase activity, but the rubber molecular weight was not always changed. Laibach et al. reported a TbREF playing critical roles in rubber particle biogenesis and rubber production in *T. brevicorniculatum* [75]. Its knockdown mutants exhibited significantly reduced rubber content correlating with lower TbCPT protein levels and less TbCPT activity in the latex, while rubber molecular mass and the stability of rubber particles were unaffected, indicating TbREF is an important component of the rubber synthetic machinery [75]. Priya et al. demonstrated that the abundance of REF mRNA transcripts in latex was 3- to 5-fold higher in high-yielding rubber tree clones than in low yielders, implying a positive correlation between REF gene expression pattern and latex yield [76]. TkSRPP3 RNAi lines showed significant decreases in root rubber content and produced dramatically lower molecular weight rubber than the control line, while TbSRPP RNAi lines showed 40–50% reduction in dry rubber content, but neither the rubber average molecular weight nor the polydispersity of the rubber was affected [47,77]. In lettuce (*Lactuca sativa*), knockdown of LsSRPP4/LsSRPP8, which account for more than 90% of total transcripts of REF/SRPP homologs in latex, individually or simultaneously, had no effect on rubber content, molecular weight or polydispersity, suggesting that SRPP might not be a necessary component for the rubber transferase complex in lettuce [78].

Although most studies proved REF/SRPP play positive roles in rubber biosynthesis, no catalytic domain was found in these proteins. Dai et al. suggested that REF/SRPP family proteins are structural proteins with function in packaging/storage of synthesized natural rubber and stabilizing of rubber particles in *H. brasiliensis*, for all the identified REF/SRPP isoforms are acidic isoelectric point proteins which can maintain the negative charge and avoid the coagulation of rubber particles [72]. This view was also supported by the fact that the addition of REF did not activate the background activity of washed rubber particles in vitro [38]. The exact function of each REF/SRPP isoform needs to be identified by further study.

#### 2.3.3. CPT-Like/CPT-Binding Protein

CPT-Like/CPT-binding proteins (CPTL/CPTBP) are the most recently isolated proteins that are involved in rubber biosynthesis which attracts great interest. They are homologs of the human Nogo-B receptor (NgBR), and localized mainly on the ER in non-rubber-producing eukaryotes and on both ER and rubber particles in rubber-producing plants [44,48,79,80,81]. The NgBR homologs usually contain one or more transmembrane domains in the N-terminal region and a low-similarity CPT-like domain in the C-terminal half [11,38]. Although they do not have catalytic residues conserved among CPTs, the RxG motif in their C-terminal was demonstrated to be critical for prenyltransferase activity [82]. AtLEW1 (LEAF WILTING1) was reported to be required for the dolichol biosynthesis and mediates plant responses to ER stress, drought, and dark-induced senescence in *Arabidopsis* [83]. Characterization of lettuce *cis*-prenyltransferase-like 2 (LsCPTL2) suggested that LsCPTL2 is a scaffold protein tethering LsCPT3 on ER and is necessary for natural rubber biosynthesis in lettuce [48]. However, yeast-expressed LsCPTL2 and LsCPT3 alone could not synthesize high-molecular-weight natural rubber in vitro without rubber particles, indicating other components may be required for the rubber biosynthetic machinery [48]. It was also proved that the tomato SlCPT3 and its partner CPT-binding protein (SlCPTBP) interact in vivo and both of them are strictly required for dolichol synthase activity [81]. The *T. brevicorniculatum* rubber transferase activator (TbRTA) was demonstrated to be an essential component of the rubber transferase complex which interacts with TbCPTs on the surface of rubber particles [43]. Its knockdown mutants showed decreased abundance of rubber particles and eliminated rubber biosynthesis [43].

**Figure 4:**
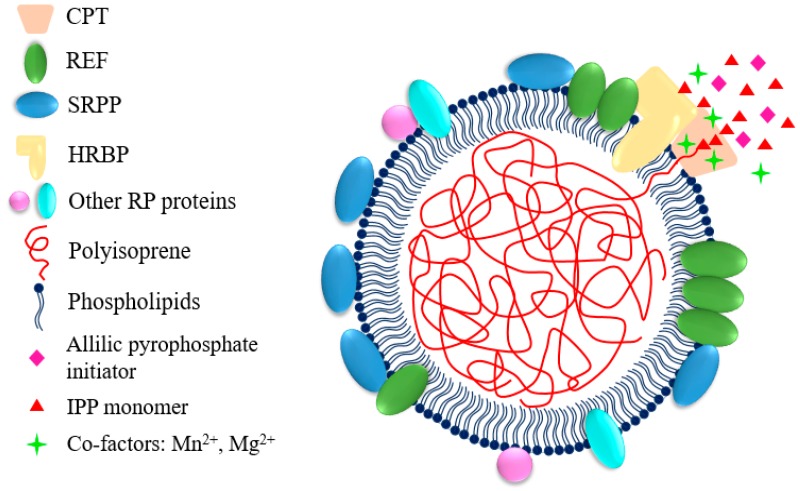
Schematic model of rubber particles. Rubber particles are made of polyisoprene, surrounded by a monolayer of lipids with proteins and other compounds. The synthesized polyisoprene chains are elongated inward and stored in the interior of the rubber particles. The *H. brasiliensis* rubber biosynthetic machinery is supposed to consist of HRT1, HRBP and REF. HRBP may work as a scaffold tethering HRT1 on the surface of rubber particles. REF may function in stabilizing the rubber biosynthetic machinery, while both REF and SRPP may play roles in rubber particle stabilization and coagulation. REF contains one predicted transmembrane domain and may be inserted deeply into the rubber particle lipidic monolayer, while SRPP has no predicted transmembrane domain and may just associate with the lipidic monolayer. CPT, *cis*-prenyltransferase; HRT1, *Hevea* rubber transferase 1; HRBP, HRT1-REF bridging protein; REF, rubber elongation factor; SRPP, small rubber particle protein; RP, rubber particle.

Yamashita et al. made progress in identifying the components of the rubber transferase complex by reconstituting rubber biosynthetic machinery on detergent-washed *H. brasiliensis* rubber particles (WRPs) in vitro [38]. HRT1-REF BRIDGING PROTEIN (HRBP), which belongs to the CPTL/CPTBP clade (Figure 3), was demonstrated to play important roles in introducing recombinant CPTs to WRPs. It interacts with HRT1/2 and REF, and may act as a bridge between HRT1 and REF to form a complex [38,44]. Co-expression of HRBP and REF decreased the amount of HRT1 which was not incorporated on WRPs, indicating they promote the incorporation of HRT1 into WRPs [38]. Introducing HRT1 onto WRPs distinctly increased the rubber transferase activity, whereas HRBP or REF alone did not activate the background activity of WRPs [38]. Co-expression of HRBP or both HRBP and REF could enhance the HRT1-derived rubber transferase activity, but had no effect on product chain length regulation [38]. Co-expression results of these proteins in *Nicotiana benthamiana* leaves showed that the HRT1/HRBP/REF complex primarily was localized on ER and ER-emerged-specific particles which supported the idea that rubber particles may originate from ER membrane [38]. Therefore, from the interaction network, rubber transferase activity assay and subcellular localization analyses, it is suggested that the natural rubber biosynthetic machinery consists of CPT, HRBP and REF [38,44] (Figure 4). As HRBP and REF have no catalytic domain or activity, they may function as structural proteins stabilizing the rubber biosynthetic machinery on rubber particles.

#### 2.3.4. Other Involved Genes/Proteins/Pathways

Genes of the MEP and MVA pathways from rubber-producing plants were cloned and functionally analyzed [84,85,86,87]. While MVA pathway was thought to be the main provider of the IPP monomer, there were different opinions about whether MEP pathway was directly involved in rubber biosynthesis. Some MEP pathway genes have high expression levels in the latex, but the seedling feeding experiment with [1-^13^C] 1-deoxy-d-xylulose triacetate, an intermediate derivative of the MEP pathway, indicated that the MEP pathway is responsible for carotenoids biosynthesis rather than rubber biosynthesis in *H. brasiliensis* latex [84]. However, in a following study, expression profiles of MEP and MVA pathway genes suggested that MEP pathway may be an alternate contributor of IPP in mature rubber trees or in clones, which do not produce a large amount of carotenoids [88]. Farnesyl diphosphate synthases (FPSs) were identified and demonstrated to be the main prenyltransferase involved in *trans*-1,4-polyisoprene (Eu-rubber) biosynthesis in *E. ulmoides* [5,6,89]. Gene co-expression network analysis showed correlated expression pattern of MEP pathway genes with *EuFPS1* and MVA pathways genes with *EuFPS5*, indicating that both MEP and MVA pathways may be involved in Eu-rubber biosynthesis [90]. However, expression patterns of MEP pathway genes were not correlated with the accumulation of Eu-rubber in fruits, while MVA pathway genes did, implying that MVA pathway rather than MEP pathway is mainly responsible for IPP supply during Eu-rubber biosynthesis in fruits [5].

## 3. Omics Analyses Provide New Insights into Natural Rubber Biosynthesis

### 3.1. Genome Analysis

Omics including genome, transcriptome, and proteome analyses of the rubber-producing plants generated high-throughput data, helping us to get a comprehensive understanding of the natural rubber biosynthesis process. Draft genome assembly of the common high-yielding *H. brasiliensis* clone RRIM 600 was firstly reported in 2013 [91] (Table 1). Subsequently, genomes of more *H. brasiliensis* cultivars were sequenced and annotated. The rubber tree is a diploid (2n = 2x = 36) with an established genome of 2.15 Gb [22]. So far, the longest assembly spans 1.55 Gb and a total of 84,440 high-confidence protein-coding genes have been predicted [22] (Table 1). The *H. brasiliensis* genome showed a striking expansion of gene families involved in natural rubber biosynthesis, revealing this might be the reason why it could produce high levels of latex [22,91,92]. Seven CPTs, 1 CPTL, 9 REFs, 8 SRPPs and multi-copy genes involved in the MVA and MEP pathways and the biosynthesis of oligomeric allylic pyrophosphates were identified and annotated in the *H. brasiliensis* genome [22,93]. Some of these rubber biosynthesis-related genes are arranged in clusters in the genome, suggesting coordinated evolution and expression for latex production [22]. *H. brasiliensis* has the highest number of disease resistance-related genes compared with other *Euphorbiaceae* genomes, indicating that these genes may play import roles in response to stresses and diseases caused by fungal pathogens [22]. Through genome or RNA sequencing, molecular markers such as simple sequence repeats (SSRs) and single nucleotide polymorphisms (SNPs) have been identified, and a high-density genetic linkage map was constructed for *H. brasiliensis* [94,95,96,97,98,99,100,101,102]. These data would be a valuable information resource for future genetic analyses such as quantitative trait loci (QTL) identification, linkage disequilibrium investigations and phylogenetic studies and a powerful tool for breeding elite rubber tree cultivars. Makita and colleagues constructed the first specific genome and transcriptome database for *H. brasiliensis* (http://matsui-lab.riken.jp/rubber/, accessed on 20 December 2018), including functional annotations and multi-transcriptome data of RNA-seq, full-length cDNAs, ESTs and genome wide transcription start sites (TSSs) [93]. Gene co-expression network is also presented in the database which provides information for identifying functionally related gene sets and genes regulated by the same transcription factor [93]. This database will accelerate our understanding of the molecular mechanism of natural rubber biosynthesis and assist both industrial and academic utilization of rubber tree and important close industrial relatives.

Genomes of other rubber-producing plants were also sequenced and assembled (Table 1). Wuyun et al. reported a high-quality assembly of ~1.2 Gb genome with at least 26,723 predicted genes for *E. ulmoides* [5]. Similar to *H. brasiliensis*, *E. ulmoides* has gene number expansion for multiple genes involved in stress responses and biosynthesis of secondary metabolites which were supposed to account for disease resistance and environmental adaptability. Five *EuFPSs* were identified in the genome. Conserved domain and phylogenetic analysis, gene expression patterns and heterogenous overexpression suggested EuFPS1, 3, and 5 may be responsible for *trans*-polyisoprene biosynthesis in *E. ulmoides*. The coordinated expression patterns of *EuSRPP1*, *2*, and *7* with the accumulation of Eu-rubber implied that they might play critical roles in Eu-rubber biosynthesis and stabilizing the rubber particle membrane in *E. ulmoides* [5]. Their results also showed that *EuFPS* and *EuREF/EuSRPP* gene families were expanded independently from the *H. brasiliensis* lineage, indicating the polyphyletic origin of polyisoprene biosynthesis in eudicots [5,89]. Lin et al. reported the genome sequencing and assembly for *T. kok-saghyz* Rodin which has a length of 1.29 Gb and contains 46,731 predicted genes [11]. The comparative studies of *T. kok-saghyz* with the non-rubber-producing plant Globe artichoke and the rubber-producing plant *H. brasiliensis* showed that the gene number is similar for enzymes in the MEP pathway and rubber initiator synthesis, but differs in the MVA pathway and rubber elongation, suggesting the MVA pathway and rubber elongation-related genes might be more important for rubber biosynthesis in *T. kok-saghyz* [11]. Only one *TkREF* was found in the *T. kok-saghyz* genome and TkREF/TkSRPPs clustered into a clade that were different from the HbREFs/HbSRPPs clade, implying genetic divergence and different mechanisms were involved in rubber biosynthesis between the two species. So far, there has been no draft genome report about *P. argentatum*, due to its complex natural ploidy series [103,104].

### 3.2. Transcriptome Analysis

Transcriptome analyses of *H. brasiliensis* laticifers or rubber particles have been investigated for more than 10 years. All of these analyses revealed that the REF/SRPP gene family members were the most abundant components of the latex transcriptome. They could account for 29% of the total ESTs [24]. Different REF/SRPP isoforms were identified and found to have tissue-specific expression patterns, indicating that they play different roles in different tissues. Unexpectedly, CPTs, which is a potential candidate for rubber polymerase, have relatively low abundance in the latex transcriptome, and not all genes in the MVA and MEP pathways could be detected among the highly expressed ESTs [24,96,105]. Expression profile analyses of genes involved in rubber biosynthesis exhibit tissue-specific TSSs, implying alternative means of transcriptional regulation besides changes in mRNA abundance [22,106]. Stress-related genes, such as REF-like proteins (HbRLPs) and ASR-like proteins (HbASRLPs)-encoding genes, also have high expression levels in the latex, which is consistent with the expansion of the stress/resistance-related genes in the genome, supporting the view that the physiological roles of natural rubber biosynthesis may be related with the defense and resistance process [22,24,105].

Transcriptome analysis provided new insights into understanding of the molecular mechanisms underlying high yield of different rubber tree clones. Tang et al. performed a comprehensive cDNA-AFLP transcript profiling on the latex of a super-high-yielding rubber tree clone SY107 and its average counterparts [8]. Their results showed that the improved sucrose loading capability, rubber biosynthesis-preferred sugar utilization, enhanced general metabolism and timely stress alleviation, rather than the rubber-biosynthesis pathway genes, are correlated with the super-high-yielding phenotype of SY107 [8]. Li et al. compared and analyzed two rubber tree varieties (RRIM 600 and RY 7-20-59) systematically, and suggested that the higher natural rubber yield in RY 7-20-59 likely results from increased total IPP supply, more IPP distribution for rubber biosynthesis and upregulation of the genes involved in rubber biosynthesis pathway [107].

The natural rubber biosynthesis regulation related transcription factors and the transcriptomes of ethephon or jasmonates-treated *H. brasiliensis* cultivars were also investigated. Aoki et al. identified the laticifer-specific genes and their promoter regions from *H. brasiliensis* [108]. They found seven common motifs consisting of eight bases exist in the 5′-upstream regions of these laticifer-specific genes through promoter clone and in silico analysis. They also identified the putative transcription factors including MYB, Zinc Finger, bHLH, bZIP, PHD Finger and NAC Domain-containing family members, which paved the way to discover the regulatory mechanisms of natural rubber metabolism and accelerated our understanding of the gene regulatory network in *H. brasiliensis* laticifer [108]. Wang et al. identified and characterized the MYB family members in the laticifer cells of *H. brasiliensis* and revealed that HblMYB19 and HblMYB44 could increase the activity of the promoters of *HbFDPS1*, *HbSRPP*, and *HRT1*, which are associated with the biosynthesis of natural rubber [109]. Sequencing and comparison of rubber tree clone CATAS8-79 and PR107 latex at the transcriptome level revealed that the endogenous jasmonates level, carbohydrate transport and metabolism, and *HMGR* and *HRT* expression levels are important for latex regeneration while the endogenous ethylene level, cellulose and lignin content of laticifer cell wall, antioxidants and glucanases are important for the duration of latex flow [110,111]. Transcriptomes analysis of *H. brasiliensis* PR107 treated with ethephon revealed that the upregulation of the key genes in the glycolytic pathway and Calvin cycle, instead of rubber biosynthesis, might be responsible for ethylene-induced latex production in rubber trees [112].

Multiple genes involved in stress responses and secondary metabolites biosynthesis exhibit high expression levels in *E. ulmoides* [5]. Libraries of stem tissues were established and major latex protein (MLP)-encoding and metallothionein-encoding genes were found to be the large parts which may have a protective role and required for homeostasis in stem tissues [6]. MEP and MVA pathway genes and rubber particle membrane protein-encoding genes were identified in *E. ulmoides*. Among them, five *trans*-isoprenyl diphosphate synthase (EuTIDS/EuFPS)-encoding genes were obtained with EuTIDS2 and 4 could complement FPP synthase-deficient in yeast while EuTIDS1, 3 and 5 were supposed to be the long-chain *trans*-polyprenyl diphosphate synthases [6]. For *P. argentatum*, because rubber synthesis is associated with the moderately cold night temperatures, transcriptome of bark tissue of the cold-acclimated guayule was investigated [64]. Stress-related protein-encoding genes were found to be the vast majority of ESTs, while transcripts-encoding proteins associated with rubber particles (such as CPTs and SRPPs) or the isoprenoid pathway enzymes that make the precursors for rubber biosynthesis (MVA enzymes and FPS) were not enriched in the cold-acclimated guayule, indicating post-translational modifications instead of gene expression control is more important for regulating enzymatic activity of the rubber transferase complex in guayule [64]. In *T. kok-saghyz*, MVA pathway key genes such as *TkHMGR1* and *TkHMGR2* and rubber elongation-related genes (*TkCPT1*, *TkCPT2*, *TkCPTL1*, and *TkSRPPs*) have high expression levels in latex and roots, suggesting that these genes may play critical roles in natural rubber biosynthesis [11]. Luo et al. reported the comparative transcriptome analysis of high and low rubber-yielding *T. kok-saghyz* genotypes and found 21,036 SNPs and 158 significantly differentially expressed transcripts [113]. However, the rubber biosynthesis-related genes, such as *CPTs*, *SRPPs* and *REFs*, were not present in the differentially expressed transcripts, implying that other regulatory mechanisms or factors may be involved in rubber yield regulation in *T. kok-saghyz*. Cao et al. performed the transcriptome analysis of MeJA-induced *T. kok-saghyz* seedling and found expression of *HMGR*, *FPS*, *IDI*, *GGPS*, *REF*/*SRPP* and certain transcription factors-encoding genes were induced by MeJA treatment [114]. *HMGR* expression was upregulated in *DREB*-overexpressing lines, suggesting JA regulates secondary metabolism and rubber biosynthesis through the interaction of JA-related TF with the genes required for rubber biosynthesis [114].

### 3.3. Proteome Analysis

Proteomics is an efficient tool to study the protein abundance and distribution in an organism [115]. Consistent with the genome and transcriptome analyses, proteome analyses also showed that REF/SRPP proteins are the most abundant components in *H. brasiliensis* latex proteome, while other high or medium-abundance proteins found were more or less different, depending on materials used and protein extraction and treatment/analytic methods [21,37,39,41,115,116]. Xiang et al. performed proteome analysis of LRPs and SRPs of *H. brasiliensis* and gave a wealth of information of protein profiles difference between them. Their results revealed that REF (14.7 kDa) was almost equally expressed on both LRPs and SRPs, while REF (19.6 kDa) was highly expressed on LRPs compared with SRPs, indicating that different isoforms of REF may play different roles on LRPs and SRPs and the REF (19.6 kDa) might play a termination role in rubber elongation reaction on LRPs [37]. MVA pathway proteins, such as HMGS, were expressed more predominantly on SRPs than on LRPs, which is consistent with the fact that SRPs have higher activity of rubber biosynthesis than LRPs. Signal transduction-related proteins, such as phospholipase D alpha (PLDα) and ethylene response factor 2 (ERF2), were also expressed at higher levels on SRPs, suggesting the possible involvement of SRPs in the signal transduction of plant hormones or stress responses [37]. Defense and stress-responsive proteins were also a kind of abundant components of the latex proteome. Heat shock cognate 70-kDa protein (HSC70) and eukaryotic translation initiation factor 5A isoform IV (eIF5A-4) had higher expression levels on SRPs, indicating that these proteins may be required for SRPs to maintain homeostasis in regulation of protein quality during rubber biosynthesis and the development of SRPs. HbASRLP1 and HbRLP1 had higher expression levels on LRPs, suggesting that LRPs might be critical in defense or stress-related responses [37]. Besides, higher abundance of β-1,3-glucanase was detected on LRPs than on SRPs, implying that LRPs play more important roles in the latex coagulation mediated by β-1,3-glucanase than SRPs [37]. Dai et al. carried out an in-depth proteome analysis of the *H. brasiliensis* rubber particles and identified 186 rubber particle proteins [39]. Besides the highly abundant REF/SRPP proteins, many proteins were firstly identified to be on the rubber particles, including the cyclophilin, cytochrome P450, small GTP-binding protein, clathrin, eukaryotic translation initiation factor, annexin, ABC transporter, translationally controlled tumour protein and ubiquitin-conjugating enzymes, which might be involved in rubber biosynthesis, rubber particle biogenesis and other biological processes [39].

Proteome of ethephon or jasmonates-treated *H. brasiliensis* cultivars and other rubber-producing plants were also investigated. Wang et al. performed the first in-depth comprehensive proteomics analysis of rubber latex following ethylene stimulation and obtained abundant proteins, ethylene responsive latex proteins and differentially expressed phosphoproteins through different analytic methods [117]. They found that ethylene improved the generation of SRPs whereas most genes involved in rubber biosynthesis were inhibited by exogenous ethylene. Their results suggested the improved latex production is probably due to the inhibition of rubber particle aggregation and the resulted prolongation of latex flow [117]. In addition, they also found that post-translational modification and isoform-specific phosphorylation might be important for ethylene-stimulated latex production [117]. Dai et al. treated *H. brasiliensis* clone Reyan 7-33-97 with ethrel and methyl jasmonate (MeJA), respectively, and identified a total of 101 latex proteins that were regulated by ethrel and/or MeJA [118]. They found that proteins required for latex regeneration, including phosphoenolpyruvate carboxylase (PEPcase), acetyl-CoA C-acetyltransferase (ACCT) and REF isoforms, and those associated with latex flow, such as chitinase and a sieve element occlusion protein, were affected by the application of ethrel, while chitinase and polyphenol oxidase were regulated by MeJA [118]. Wahler et al. carried out a comprehensive analysis of the latex proteome of *T. brevicorniculatum*, and obtained 278 unique proteins [40]. Their study revealed that SRPPs were the most abundant proteins in the rubber particle subproteome, and proteins involved in rubber biosynthesis were distributed among different fractions of the latex proteome [40]. However, proteins involved in lipid metabolism and transport rather than in stress responses were enriched in the latex proteome, suggesting different metabolic activities of latex in different plant species [40].

## 4. Possibility of Natural Rubber Biosynthesis In Vitro or in Genetically Engineered Microorganisms

Identification of the synthetic or regulatory genes involved in natural rubber provides useful information for improving rubber yield in rubber-producing plants. For example, overexpression of an *EuIPI* showed 3- to 4-fold increase of *trans*-polyisoprene content in transgenic lines of *E. ulmoides* [119]. However, for perennial woody plants such as *H. brasiliensis*, it would take years of growth to see the phenotype. Although more plants species have been investigated and domesticated for industrial rubber production purposes, the plant-derived rubber production and harvest is a time-consuming and labor-intensive process. The rubber-producing plants need land to be planted and usually have a relatively long mature period. Their growth also depends on climate and might be disrupted by diseases and pests.

Is it possible to synthesize rubber in vitro or in genetically engineered microorganisms? So far, almost all IPP incorporation assays and CPT/REF/SRPP activity assays have used rubber particles or latex washed bottom fraction as an essential component for investigating rubber synthesis in vitro, which indicated the indispensability of rubber particles in rubber synthesis. Yamashita et al. introduced the cell-free wheat germ system-expressed HbHRT1 or LsCPT3 to the detergent-washed rubber particles separately [38]. Both recombinant HbHRT1 and LsCPT3 showed rubber transferase activity and synthesized rubber was obtained in vitro. They also revealed the formation of the protein complex consisting of HRT1, REF and HRBP which functions as the natural rubber biosynthetic machinery on rubber particles [38]. However, the study could not eliminate the possibility that some unknown proteins that persist on the detergent-washed rubber particles have rubber transferase activity or work as an indispensable component in the rubber transferase complex. Oil bodies share similar properties with rubber particles [4]. It is possible to prepare artificial oil bodies with triacylglycerol, phospholipids, and oleosins which can be used to purify recombinant proteins fused with oleosins [120,121]. Reconstituting rubber particles using natural rubber, phospholipids, and recombinant rubber transferase candidates such as CPT/REF/SRPP/CPTL isoforms would reveal the minimum structure unit of the rubber synthetic machinery. However, the cost price of substrates like FPP and IPP might be a stumbling block to the synthesis of natural rubber in vitro.

**Figure 5:**
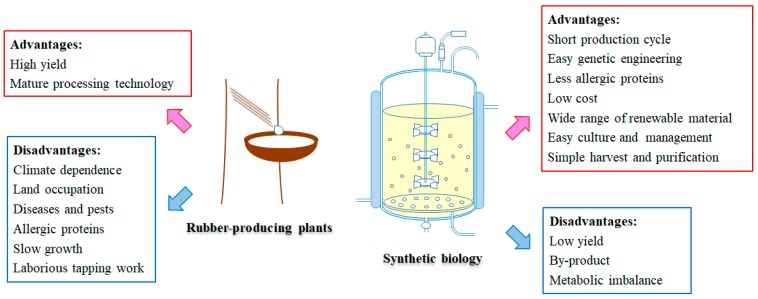
Comparison of rubber production from rubber-producing plants (take *H. brasiliensis* for example) and synthetic biology.

Compared with the in vitro system, synthesis of natural rubber by fermentation of engineered microorganisms from renewable sugar exhibits more advantages (Figure 5). The biosynthesis of a mass of basic compounds with relatively low molecule weight has been achieved in engineered microorganisms including monomers for synthetic rubber, such as isoprene and styrene [122,123]. Synthesis of some biopolymers such as polyhydroxyalkanoic acids (PHAs) which consist of a wide range of different HAs was also found in many bacteria [124]. Therefore, is it possible to synthesize rubber in engineered microorganisms? As the biogenesis of rubber particles was supposed to occur in ER or Golgi and the activity of rubber transferase might be modified by the eukaryotic post-translation modification, the endomembrane-containing eukaryotic organism would be a suitable host for ectopic rubber biosynthesis. Oleaginous yeasts, such as *Yarrowia lipolytica* or *Cryptococcus curvatus*, may be ideal candidates, because they are able to accumulate up to about 50% (dry weight) of storage carbohydrates in oil bodies which resemble the rubber particles [4,125]. Researchers suggested that disruption of the isoprenoid branch pathway *squalene synthase* (*SQS*/*ERG9*) gene might lead to a considerable increase in the flow of substrates to rubber biosynthesis [124]. As the outline of the natural rubber biosynthesis machinery becomes clearer, it seems we went one step further for the natural rubber biosynthesis in engineered eukaryotic cells. However, transient co-expression of the suggested natural rubber biosynthesis complex HRT1–HRBP–REF in *N. benthamiana* leaves did not lead to the formation of rubber particles, indicating that other components may be involved in natural rubber biosynthesis or rubber particle biogenesis [38].

## 5. Conclusions and Perspectives

As HRT1, REF and HRBP were identified to be components of the rubber transferase complex, the outline of the natural rubber biosynthetic machinery is becoming clearer. However, the exact role of each component, other proteins involved in rubber biosynthesis, rubber particle biogenesis and rubber molecular weight determination and the corresponding mechanisms remain elusive. To reveal the above mysteries, omics will continue to be a powerful tool to find indispensable components of rubber transferase complex and the regulatory factors of natural rubber biosynthesis. Other advanced technologies such as CRISPR would accelerate the identification of key regulatory or catalytic genes and the improvement of the high-yield variants. Reconstruction of functional artificial rubber particles in vitro will enrich our knowledge of rubber particle biogenesis and rubber transferase assembly, and pave the way for the non-plant-derived natural rubber production.

## Figures and Tables

**Table 1:** Summary of genome assembly of the main rubber-producing plants.

Species	Chromosome Number	Assembly Length/Estimated Length	Predicted Gene Number	BioProject/Accession No.	Ref.
*Hevea brasiliensis* RRIM 600	2N = 2X = 36	1.1 Gb/2.15 Gb	68955 8 CPTs, 10 SRPPs, 12 REFs	GenBank: AJJZ01000000	[91]
*Hevea brasiliensis* Reyan 7-33-97	2N = 2X = 36	1.37 Gb/1.46 Gb	43792 11 CPTs, 8 REFs, 10 SRPPs	GenBank: LVXX01000000	[92]
*Hevea brasiliensis* RRIM 600	2N = 2X = 36	1.55 Gb/2.15 Gb	84440 7 CPTs, 1 CPTL, 9 REFs, 8 SRPPs	GenBank: PRJDB4387	[22]
*Taraxacum kok-saghyz* line 1151	2N = 2X = 16	1.29 Gb/1.4 Gb	46731 9 CPTs, 2 CPTLs, 1 REF, 9 SRPPs	Genome Warehouse: PRJCA000437 GWHAAAA00000000	[11]
*Eucommia ulmoides* a wild *E. ulmoides* tree in Shennongjia	2N = 2X = 34	1.2 Gb/1.1 Gb	26723 5 FPSs, 5 REFs, 7 SRPPs	Genome Warehouse: PRJCA000677 GWHAAAL00000000	[5]

CPT, *cis*-prenyltransferase; CPTL, CPT-Like protein; FPS, Farnesyl diphosphate synthase; REF, rubber elongation factor; SRPP, small rubber particle protein.
